# Breast Fibroblasts and ECM Components Modulate Breast Cancer Cell Migration through the Secretion of MMPs in a 3D Microfluidic Co-Culture Model

**DOI:** 10.3390/cancers12051173

**Published:** 2020-05-06

**Authors:** Karina M. Lugo-Cintrón, Max M. Gong, José M. Ayuso, Lucas A. Tomko, David J. Beebe, María Virumbrales-Muñoz, Suzanne M. Ponik

**Affiliations:** 1 Department of Biomedical Engineering, University of Wisconsin, Madison, WI 53705, USA; km.lugo12@gmail.com (K.M.L.-C.); djbeebe@wisc.edu (D.J.B.); 2 Department of Pathology and Laboratory Medicine, University of Wisconsin, 1111 Highland Ave., Madison, WI 53705, USA; ayusodomingu@wisc.edu; 3 University of Wisconsin Carbone Cancer Center, Wisconsin Institutes for Medical Research, 1111 Highland Ave., Madison, WI 53705, USA; gongm@trine.edu; 4 Department of Biomedical Engineering, Trine University, Angola, IN 46703, USA; 5 Department of Cell and Regenerative Biology, University of Wisconsin, Madison, WI 53705, USA; ltomko@wisc.edu

**Keywords:** ECM composition, breast Cancer, 3D collagen, microfluidics, organotypic, fibronectin

## Abstract

The extracellular matrix (ECM) composition greatly influences cancer progression, leading to differential invasion, migration, and metastatic potential. In breast cancer, ECM components, such as fibroblasts and ECM proteins, have the potential to alter cancer cell migration. However, the lack of in vitro migration models that can vary ECM composition limits our knowledge of how specific ECM components contribute to cancer progression. Here, a microfluidic model was used to study the effect of 3D heterogeneous ECMs (i.e., fibroblasts and different ECM protein compositions) on the migration distance of a highly invasive human breast cancer cell line, MDA-MB-231. Specifically, we show that in the presence of normal breast fibroblasts, a fibronectin-rich matrix induces more cancer cell migration. Analysis of the ECM revealed the presence of ECM tunnels. Likewise, cancer-stromal crosstalk induced an increase in the secretion of metalloproteinases (MMPs) in co-cultures. When MMPs were inhibited, migration distance decreased in all conditions except for the fibronectin-rich matrix in the co-culture with human mammary fibroblasts (HMFs). This model mimics the in vivo invasion microenvironment, allowing the examination of cancer cell migration in a relevant context. In general, this data demonstrates the capability of the model to pinpoint the contribution of different components of the tumor microenvironment (TME).

## 1. Introduction

Breast cancer is among the three most common cancers worldwide and is the most common malignancy in women [1,2]. Currently, cancer metastasis accounts for 90% of cancer-related deaths. Cancer metastasis is a multistep process by which tumor cells migrate from the primary tumor through the surrounding matrix, intravasate into the vasculature (either blood or lymphatic), extravasate, and colonize a distant organ [3]. In order to metastasize, cancer cells must degrade the surrounding ECM to invade and migrate through the stroma. Within the surrounding matrix, cancer cells interact with biochemical and biophysical components of the matrix (e.g., matrix composition) as well as stromal components, such as fibroblasts, that regulate metastatic outcome [4]. Despite significant advances in the understanding of breast cancer, metastasis remains a poorly understood process and is the main cellular event leading to poor patient survival. Therefore, it is essential to understand the migration of cancer cells within the stroma to develop successful strategies that target metastasis.

The tumor microenvironment (TME) has emerged as a critical player in cancer progression and metastasis [5,6,7]. The TME has two major components, cellular and a non-cellular component [8,9]. The cellular component comprises malignant and non-transformed cells, including fibroblasts, immune cells, vasculature, and adipocytes [10,11]. Among the many components of the TME, fibroblasts have been reported as the major cellular component that regulates cancer cell progression due to the ability of cancer cells to drive fibroblasts into an activated phenotype [12,13,14]. In breast cancer, fibroblasts have been shown to promote tumor growth, migration, and metastasis [15]. Fibroblasts can promote tumor progression by remodeling the extracellular matrix (ECM) structure and composition. The ECM is the non-cellular component of the TME, and it is mostly composed of collagen I, which has been demonstrated to align next to the boundary of the tumor, thereby facilitating cancer cell migration [16,17,18]. In addition, recent in vivo proteomics data revealed that breast tissue of invasive ductal carcinoma patients had increased deposition of other components of the ECM (e.g., fibronectin) as compared to normal tissues [19]. Furthermore, areas of collagen alignment surrounding the tumor correlated with increased levels of FN, TNC, TSP2, and Col12. Clinically, the increase in fibronectin expression has been associated with poor clinical outcomes, decreased survival rates, and therapeutic resistance [20,21]. Although it is evident then that the TME plays a crucial role in tumor progression and metastasis, critical TME components, such as fibroblasts and alterations in ECM composition, are not included in traditional in vitro assays that investigate cancer cell migration.

Traditional in vitro models for studying cell migration include wound healing assays and transwell assays, and present several limitations [22,23]. For example, studies using wound healing assays are performed in two-dimensional substrates (2D). However, it is well known that the mechanisms of cell migration used in 2D differ from those in 3D environments [24,25]. While transwell assays have been used to study cell migration in 3D environments, they are designed for endpoint analysis of vertical cell invasion through a thin layer of 3D matrix [26,27]. Another limitation from traditional in vitro cell migration assays is the lack of additional cell types from the TME, which are known to have an impact on cancer cell migration [28]. Recently, a better recapitulation of the TME has been demonstrated using 3D microenvironments [29] and microfluidic platforms [30,31,32,33]. In the context of breast cancer migration, microfluidic models have been developed to co-culture cancer cells with components, such as blood vessels [34], lymphatic vessels [35] and immune cells [36]. Several studies have focused on the interaction between cancer cells and fibroblasts in breast cancer migration and progression [37,38], and the interactions of these cells that recapitulate the stromal activation that occurs during tumor invasion [39]. As for the ECM, microfluidic models have investigated the migration of cancer cells in 3D collagen matrices [40] and have changed parameters of the ECM, such as density [41] and composition [42]. However, most studies have focused on studying only one parameter at a time, either the matrix or the stromal composition, therefore limiting our understanding of more complex microenvironments.

In this work, we incorporated specific TME components (i.e., fibroblasts and a fibronectin-rich matrix) in a relevant 3D platform to build the complexity of the TME. Specifically, we mimicked an invasive stage of breast cancer surrounded by a complex and relevant TME using a microfluidic platform previously developed in our laboratory. This platform enables the creation of a lumen structure within a 3D collagen matrix, which is easily modified to include different fibroblasts and ECM compositions. Based on in vivo proteomics literature [19,43,44], we chose to assess the effects of fibronectin within a collagen matrix, in addition to either human mammary fibroblasts (HMFs) or cancer-associated fibroblasts (CAFs), which were embedded into the matrix. In this setup, we added metastatic breast cancer cells (MDA-MB-231) into the lumen structure to recreate a tumor-like mass invading the surrounding stroma. Then, we assessed their migration distance and matrix remodeling, as well as the crosstalk with HMFs or CAFs. Overall, in this paper, we used previously reported ECM proteomics data to recapitulate tumor-like conditions in vitro and understand how different ECM compositions, matrix, and fibroblast composition, impact the migration of breast cancer cells. Finally, we discuss the effects of TME components in potential therapeutic options (i.e., MMP inhibitors). To our knowledge, this is the first report of a microfluidic device being used to screen the effects of TME components on cancer cell migration and TME effects on the efficacy of cell migration inhibitors.

## 2. Results

### 2.1. In Vitro 3D Microfluidic Model of the Breast Microenvironment and Characterization of the Cellular Components of the Model

**Figure 1:**
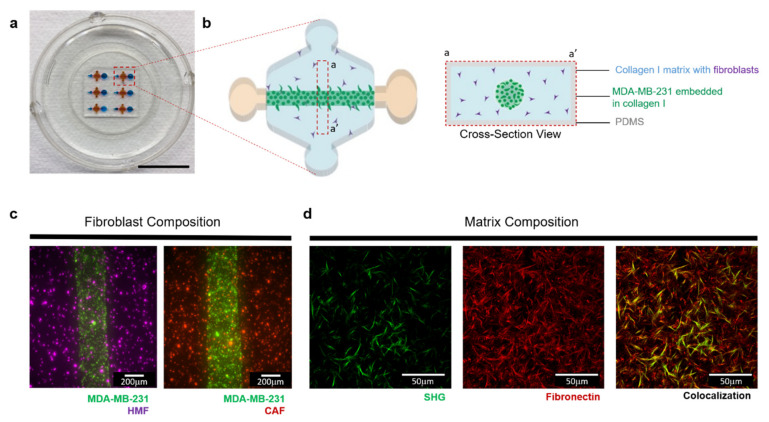
3D co-culture model that recapitulates different tumor microenvironments. (**a**) Photograph of the co-culture model (Scale bar = 10 mm). (**b**) Schematic magnification of the device, top view (left) and cross-section view (right), showing the basic components of the microenvironment that are included in the model. The matrix and embedded fibroblast composition are tailored to mimic different microenvironments. (**c**) Top view image showing MDA-MB-231s (green) co-cultures with normal fibroblasts (purple) or cancer-associated-fibroblasts (red) 1h after seeding. (**d**) Collagen I matrix is supplemented with 100 μg/mL fibronectin to mimic a tumor-like microenvironment. Visualization of the matrix was performed where collagen fibers are shown in green and were visualized by Second Harmonic Generation imaging (left). Immunofluorescence staining was performed on gels to detect the presence of fibronectin, as shown in red (middle). The composite image shows some fibers overlapping (right).

Using a previously described microfluidic called LumeNEXT, we sought to mimic an invasive stage of breast cancer and investigate the impact of ECM and stromal cell interactions on cancer cell invasion (Figure 1a). Using this device, the lumen was filled with a solution of collagen-containing metastatic breast cancer cells (MDA-MB-231) surrounded by a collagen matrix with fibroblasts (Figure 1b), recapitulating the scenario of a tumor mass invading the stroma. The surrounding matrix was then tailored to incorporate normal mammary fibroblasts or cancer-associated fibroblasts (referred to as HMFs and CAFs throughout the text) (Figure 1c), allowing for cancer-stromal crosstalk. In addition, to determine the influence of the matrix composition in cancer cell migration, we incorporated fibronectin (FN) with the collagen solution before polymerization. The successful incorporation of FN in the collagen was validated by immunofluorescence staining (Figure 1d), as seen in red, and the collagen fibers were visualized by second harmonic imaging (SHG).

We also performed a characterization of the cells used for this model. First, we confirmed the expression of GFP in 97% ± 2.3% of the MDA-MB-231s (Figure S1a). In addition, these cancer cells were stained using F-actin (red) to assess their morphology, revealing their characteristic invasive phenotype (Figure S1a). Then, we performed a podoplanin staining in both fibroblast populations to corroborate the activation of the CAFs, which revealed positive staining for the CAFs but not the HMFs (Figure S1b). In addition, CAFs are known to have distinct functional features from their normal counterparts, such as distinct cytokine secretion profiles and deposited ECM. Therefore, to point out the differences between these fibroblast populations, conditioned media was collected for analysis of soluble factors via MAGPIX. The results revealed a distinct cytokine and pro-inflammatory profiles between these cells (Figure S1c,d). Specifically, CAFs have significantly higher levels of IL-6 and VEGF-C and significantly lower levels of PDGF-B and CXCL12. Finally, we wanted to investigate the deposition of FN of the cells. For this, ECM-targeted mass spectrometry analysis was performed, revealing a higher deposition of fibronectin by the CAFs (Figure S1e).

To characterize the interaction between the fibroblasts and cancer cells, we used a custom co-culture 96-well plate (MicroDUO-Onexio Biosystems, LLC, Madison, WI, USA). The use of this plate allows the formation of a liquid bridge between adjacent wells, connecting the two cells populations cultured in each well via soluble factors (Figure S2a). Briefly, both cell populations (i.e., MDA-MB-231 and HMFs or CAFs) are seeded in each well and are cultured for 24 h to allow cell attachment. After that, media was added, forming the liquid bridge between the wells and allowing the co-culture for 48 h. Then, we assessed the growth rate of the cancer cells via GFP intensity reading. Interestingly, the results showed a significant increase in growth rate in co-culture with HMFs as compared to the MDA-MB-231 monoculture and co-culture with CAFs (Figure S2b). Next, we evaluated the nuclei count of the fibroblast in monoculture and co-culture with MDA-MB-231 to determine the influence of cancer cells in the growth of fibroblasts. The results showed an increase in nuclei count in the HMFs upon co-culture with MDA-MB-231s as compared to HMFs monoculture (Figure S2c). On the other hand, the results showed similar nuclei count in both conditions, CAFs co-culture with MDA-MB-231, and CAFs monoculture (Figure S2d). Therefore, we hypothesize that the crosstalk of soluble factors between MDA-MB-231 is leading to the activation of the HMFs, consistent with many literature reports [45,46]. Thus, we sought to evaluate the activation of the fibroblasts via fibroblast activation protein (FAP) staining [47]. The results showed a positive FAP staining in the HMFs co-cultured with MDA-MB-231s but negative staining when HMFs were cultured in monoculture (Figure S2e). Therefore, our results demonstrate that cancer cells are activating these fibroblasts. On the other hand, the results showed a positive FAP staining in the CAFs co-culture and monoculture (Figure S2f) as expected, given that these fibroblasts have been characterized as activated fibroblasts.

### 2.2. Influence of ECM Protein and Fibroblast Composition on Breast Cancer Cells Migration

**Figure 2:**
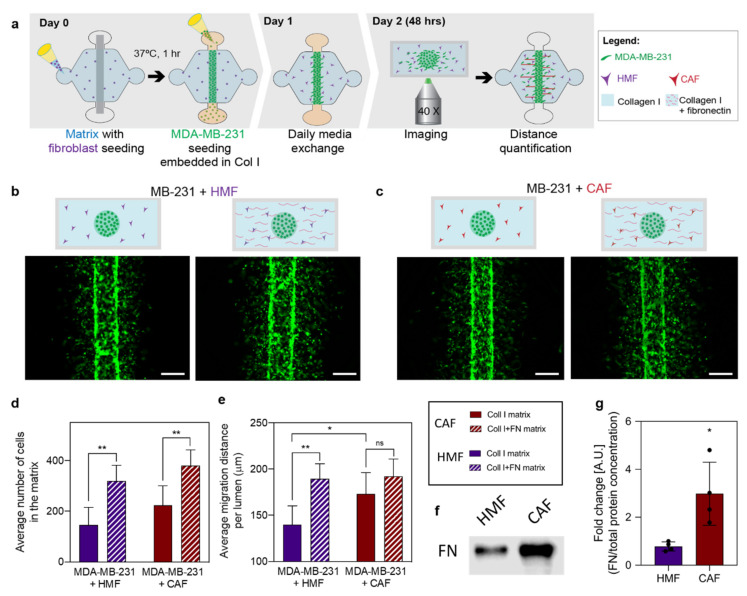
Influence of extracellular matrix (ECM) protein and fibroblast composition in cancer cell migration. (**a**) Schematic of the experimental process consisting of cell seeding, media exchanges, and imaging after 48 h of culture to track cell migration. (**b**,**c**) Fluorescence images of green fluorescent protein (GFP) tagged MDA-MB-231s within different matrix compositions in co-culture with human mammary (HMFs) and cancer-associated fibroblasts (CAFs). (**b**) MDA-MB-231 co-cultures with HMFs in a collagen matrix (left) and a fibronectin-rich matrix (right). (**c**) MDA-MB-231 co-cultures with HMFs in a collagen matrix (left) and a fibronectin-rich matrix (right). Scale bar = 200 µm. (**d**) The average number of cells in the matrix. (**e**) Average migration distance measured from the edge of the lumen after 48 h of culture. (**f**) Representative western blot of fibronectin (**g**) Quantification of fibronectin protein normalized to total protein determined by SYPRO Ruby staining (whole lane fluorescence). Bars represent average ± SD, n = at least four individual devices. * *p* ≤ 0.05, ** *p* ≤ 0.01.

After the cell characterization, we sought to investigate the effect of a fibronectin-rich matrix and fibroblasts on cancer cell migration in a more relevant microenvironment. To this end, cancer cells were seeded in the different matrix compositions (i.e., fibronectin-rich collagen or collagen alone) with embedded fibroblasts (i.e., CAFs or HMFs) for 48h (Figure 2a–c). Cancer cells stably expressing GFP were visualized in all four conditions to analyze the number of migrating cancer cells and the migration distance from the edge of the lumen using Image J. Qualitatively, an increase in the number of migrating cells was observed in a fibronectin-rich matrix as compared to the collagen only matrix, regardless of the type of fibroblasts present (Figure 2b,c). In the presence of HMFs, the average number of migrating cells was 146 ± 70 cells for the collagen matrix and 319 ± 62 cells for the fibronectin-rich matrix, revealing a significant increase in the number of migrating cells within a fibronectin-rich matrix (** *p* = 0.0019) (Figure 2d). In the presence of CAFs, the average number of migrating cells was 224 ± 76 cells for the collagen matrix and 380 ± 61 cells for the fibronectin-rich matrix, revealing a significant increase in the number of migrating cells within a fibronectin-rich matrix (** *p* = 0.0063) (Figure 2d). When comparing the influence of HMFs and CAFs in the number of migrating cancer cells, no differences were found. Qualitatively, changes in cell migration distance were observed (Figure 2b,c). In the presence of HMFs, the average migration distance was 139.9 ± 20.4 μm for the collagen matrix and 189.6 ± 16.3 μm for the fibronectin-rich matrix, revealing a significant increase in the migration distance through a fibronectin-rich matrix (** *p* = 0.0015) (Figure 2e). However, in the presence of CAFs, the average migration distance was 173.2 ± 23.2 μm for the collagen matrix and 192.3 ± 18.7 μm for the fibronectin-rich matrix, revealing no differences in the migration distance within the different matrices (Figure 2e). When comparing the influence of HMFs and CAFs in the cancer cells’ migration distance, a significant increase was found in the presence of CAFs within a collagen matrix (* *p* = 0.0365), compared to HMF. To determine whether CAFs secrete more fibronectin than HMFs, the expression of fibronectin in CAFs and HMFs cultured in 3D collagen matrices was assessed via Western blot. As anticipated, fibronectin expression was significantly increased in CAFs as compared to HMFs (Figure 2f,g). Whole Western blots and densitometry readings can be found in Figure S7 and Table S1, respectively.

### 2.3. Influence of ECM Protein and Fibroblast Composition on MMPs Secretion

**Figure 3:**
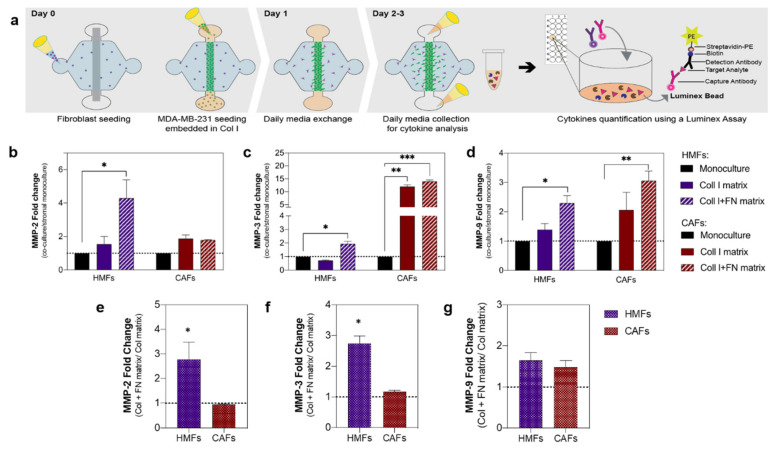
Influence of ECM protein and fibroblast composition on MMPs secretion. (**a**) Schematic of the process. Metalloproteinases (MMP) concentration for the different microenvironments was determined via a multiplex bead-based ELISA. (**b**–**d**) MMPs fold change in co-culture with HMFs (purple bars) and CAFs (red) within a collagen matrix (solid bar) and a fibronectin-rich matrix (striped bar) fibroblast monoculture showed in solid black. (**b**) MMP-2-fold change. (**c**) MMP-3-fold change. (**d**) MMP-9-fold change. (**e**–**g**) MMP fold increase in the fibronectin-rich matrix compared to the collagen-only matrix for the co-culture with HMFs (patterned purple) and CAFs (patterned red) (e) MMP-2- (**f**) MMP-3- (g) MMP-9. Bars represent average ± SD, n = at least four individual devices. * *p* ≤ 0.05, ** *p* ≤ 0.01, *** *p* < 0.001.

Due to the known relationship between cancer progression and MMPs, we next focused on studying the secretion of MMPs within the different tumor-promoting microenvironments (Figure 3a). To achieve this, we measured the secretion levels of several MMPs implicated in breast cancer progression with a multiplex magnetic bead-based ELISA (i.e., Luminex MAGPIX). All studied factors were within detectable ranges. In general, an increased level of MMPs (i.e., MMP-2, MMP-3, and MMP-9, respectively) was observed in most of the co-cultures (Figure 3b–d), compared to the fibroblast monocultures. The MMP secretions were compared to the fibroblast monoculture since the MMP levels of the cancer cell monocultures were lower (Figure S3). In the co-culture with HMFs, a significant increase in MMP-2 (4.3-fold), MMP-3 (2-fold), and MMP-9 (2.3-fold) within a fibronectin-rich matrix was observed (* *p* = 0.0351, *p* = 0.0101 and *p* = 0.0121, respectively). On the other hand, in co-culture with CAFs, a significant increase in MMP-3 was found for the collagen matrix (12-fold) and the fibronectin-rich matrix (14-fold) (** *p* = 0.0013 and *** *p* = 0.0006, respectively) and, a significant increase in MMP-9 (3-fold) within a fibronectin-rich matrix (** *p* = 0.0084) (Figure 3b–d).

In order to determine if matrix composition had an impact on cell MMP secretions, we calculated the fold change of the fibronectin-rich matrix over the collagen matrix for the different MMPs (Figure 3e–g). In the co-culture with HMFs, we found an increase in MMP-2 (2.8-fold), MMP-3 (2.7-fold) in the fibronectin-rich matrix, as compared to the collagen matrix. In contrast, matrix composition did not significantly change MMP secretion in co-cultures with CAFs.

### 2.4. Co-Culture with Fibroblasts Induces Matrix Degradation

**Figure 4:**
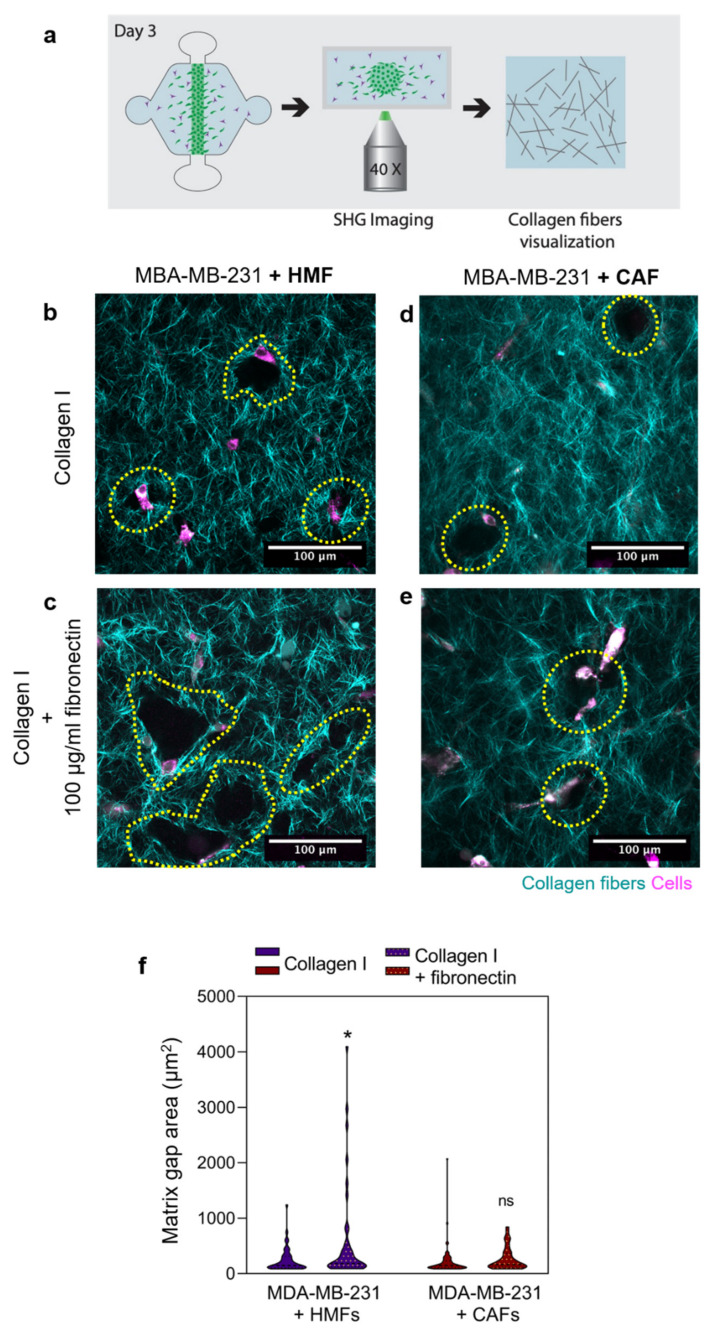
Matrix visualization via second harmonic generation (SHG) imaging. (**a**) Schematic of the process. (**b**–**e**) Collagen fibers are depicted in cyan, whereas cells appear in magenta. Collagen degradation and remodeling are observed in the form of gaps in the matrix. In co-culture with HMFs, imaging was performed in the collagen matrix (**b**) and fibronectin-rich matrix (**c**). In co-culture with CAFs, imaging was performed in the collagen matrix (**d**) and fibronectin-rich matrix (**e**). (**f**) Quantification of matrix gap area. The Violin plots represent the distribution of the data with the average and SD, n = at least four individual devices. * *p* ≤ 0.05.

Since we observed increased secretion of MMPs in the co-cultures, we sought to determine if this increase influenced matrix degradation and remodeling. To visualize the collagen fibers, Second Harmonic Generation (SHG) imaging was performed after 48 h of culture (Figure 4a). Evident matrix degradation is observed in co-cultures with HMFs and CAFs in both matrix compositions, in the form of gaps within the matrix (Figure 4b–e). Qualitatively, in co-culture with HMFs within a fibronectin-rich matrix, larger gaps were observed as compared to the collagen matrix. Conversely, in co-culture with CAFs, similar gap sizes were observed within both matrix compositions. To quantify the gap void area, image analysis was performed using ImageJ. In the presence of HMFs, the gap area was 244.0 ± 33.94 μm^2^ for the collagen matrix and 416.0 ± 40.25 μm^2^ for the fibronectin-rich matrix, revealing a significant increase in the gap area in the fibronectin-rich matrix (* *p* = 0.05) (Figure 4f). In the presence of CAFs, the gap area was 211.9 ± 27.91 μm^2^ for the collagen matrix and 265.9 ± 32.72 μm^2^ for the fibronectin-rich matrix, revealing no differences in gap area within the different matrices (Figure 4f).

The gaps observed in the matrix were indicative of matrix degradation, and only minimal gaps were observed in the MDA-MB-231 monocultures (Figure S4a,b). In the MDA-MB-231 monoculture, the gap area was 14.90 ± 0.3271 μm2 for the collagen matrix and 23.45 ± 0.6627 μm2 for the fibronectin-rich matrix, revealing a significant increase in the gap area in the fibronectin-rich matrix (* p = 0.0187) (Figure S4c). When comparing MDA-MB-231 monoculture with the co-culture with HMFs, there was a significant increase in gap size in the co-cultures (* p = 0.0462), which was not observed in the collagen alone matrix. On the other hand, when comparing MDA-MB-231 monoculture with the CAF co-culture, no differences in gap area were found.

### 2.5. Effect of MMP Inhibition in Breast Cancer Migration

**Figure 5:**
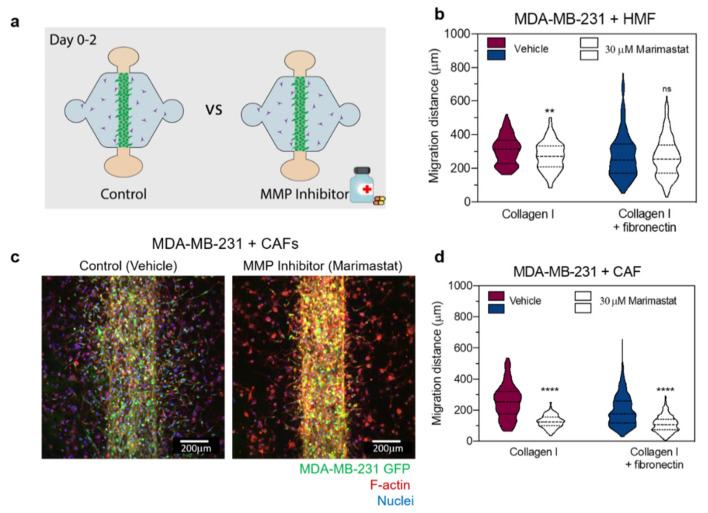
Effect of MMP inhibition on cancer cell migration. (**a**) Schematics of the experimental conditions. Co-cultures within the different matrices were treated for 48 h with 30 µM marimastat (MMP inhibitor) and a Dimethyl sulfoxide (DMSO) vehicle (control). (**b**) Migration distance of MDA-MB-231 in co-cultures with HMFs in the collagen matrix (left) and the fibronectin-rich matrix (right) for the vehicle control and treatment. (**c**) Representative image of the co-culture with CAFs for the vehicle control (left image) and treatment with 30 µM marimastat (right image). (**d**) Migration distance of MDA-MB-231 in co-cultures with CAFs in the collagen matrix (left) and the fibronectin-rich matrix (right) for the vehicle control and treatment with 30 µM marimastat. The Violin plots represent the distribution of the data with the average and SD, *n* = 3 for at least four individual devices. ** *p* < 0.01 and **** *p* < 0.0001.

To investigate if the degradation of the matrix was responsible for changes in MDA-MB-231 migration, an MMP inhibitor cocktail (marimastat) was used. All cultured conditions were treated with 30 µM marimastat or vehicle control (dimethyl sulfoxide—DMSO) for 48 h and then were imaged (Figure 5a). Then, the migration distance of the cancer cells from the edge of the lumen was quantified. In the presence of HMFs within a collagen matrix, the average migration distance was 308.3 ± 86.1 µm for the vehicle treatment and 272.8 ± 86.0 µm for the marimastat treatment, revealing a significant decrease in migration distance with marimastat treatment (** *p* = 0.0069) (Figure 5b). In contrast, HMF co-culture within a fibronectin-rich matrix, the average migration distance was 276.5 ± 135.0 µm for the vehicle treatment and 266.8 ± 117.2 µm for the marimastat treatment, revealing no differences in migration distance with marimastat treatment (Figure 5b). In co-culture experiments with CAFs, the average migration distance was 254.4 ± 109.1 µm for the vehicle treatment and 125.0 ± 40.9 µm for the marimastat treatment within a collagen matrix (Figure 5c), revealing a significant decrease in migration distance with marimastat treatment (**** *p* < 0.0001) (Figure 5d). Similarly, within a fibronectin-rich matrix, the average migration distance in the presence of CAFs was 196.1 ± 99.0 µm for the vehicle treatment and 110.3 ± 53.3 µm for the marimastat treatment, revealing a significant decrease in migration distance with marimastat treatment (**** *p* < 0.0001) (Figure 5d).

In the absence of a fibroblast enriched matrix, the migration distance of MDA-MB-231 monocultures within a collagen matrix was significantly decreased with marimastat treatment (**** *p* < 0.0001), 357.9 ± 108.9 µm for the vehicle treatment compared to 201.6 ± 67.5 µm for the marimastat treatment (Figure S5). Treatment with marimastat resulted in a similar decrease in migration distance of MDA-MB-231 monocultures within a fibronectin-rich matrix, 316.6 ± 134.3 µm for the vehicle treatment and 211.3 ± 99.5 µm for the marimastat treatment (**** *p* < 0.0001) (Figure S5). The decrease in cancer cell migration distance upon MMP inhibitor treatment points toward a lack of matrix remodeling, which hindered migration. To confirm this hypothesis, we sought to determine the influence of the MMP inhibitor in matrix reorganization by analyzing the matrix gap area via SHG imaging (Figure S6a). Interestingly, MMP inhibition did not alter the matrix gap area in co-cultures with HMFs regardless of matrix composition (Figure S6b). However, in the presence of CAFs, MMP inhibition significantly decreased the gap area for both matrices (Figure S6c).

## 3. Discussion

In recent years, the TME has emerged as a highly regulative factor of cancer progression [48]. It is well known that the ECM of the breast TME can promote cancer cell invasion, migration, and metastatic potential [49,50]. However, the few in vitro models assessing the influence of the TME components on cancer cells have been studied using 2D platforms, in which it is challenging to mimic the in vivo microenvironment accurately. 

To better understand cancer cell migration within different tumor-like microenvironments, we used a 3D microfluidic model that recapitulates different microenvironments. Using this model, we sought to investigate the effect of fibronectin-rich matrices and fibroblasts on MDA-MB-231 cancer cell migration. To generate the different stromal components of the model, we used normal (HMFs) and cancer-associated fibroblasts (CAFs). The identification of the cancer-associated phenotype was corroborated via podoplanin staining [51]. The staining was negative for the HMFs and positive for the CAFs, demonstrating that CAF have an activated phenotype that is associated with poor prognosis in breast cancer [52]. In addition, we assessed the soluble factors and the amount of fibronectin (FN) these fibroblasts deposited. This analysis revealed that both fibroblast populations have distinct cytokines and pro-inflammatory secretion profiles. For example, CAFs secrete higher amounts of IL-6, VEGF-C, and CCL2, which is distinctive of these cells [53]. Interestingly, we found higher amounts of CXCL12 in HMFs, which is usually found in activated fibroblasts [54]. Finally, we found that CAFs deposit more FN, which is in agreement with previous literature [55]. These results indicate that we are using populations of fibroblasts that represent a normal and tumor-like microenvironment.

In addition, we sought to characterize the interactions among the cancer cells and fibroblasts via soluble factor crosstalk. This characterization revealed that cancer cells crosstalk with HMFs resulted in an increase in MDA-MB-231s growth rate. Interestingly, these results are in agreement with recent literature demonstrating that normal breast fibroblasts that secrete high amounts of CXCL12, which is characteristic of our HMFs, induce MDA-MB-231 proliferation via the CXCR4 receptor [56]. On the other hand, we found an increase in nuclei counts in the HMFs co-cultured with the MDA-MB-231s. Overall, these results demonstrate the complexity of the interactions between these cells and could point out the capability of the cancer cells to induce changes in normal fibroblasts (e.g., activation of fibroblasts). Thus, we decided to evaluate the activation of the fibroblasts via fibroblast activation protein (FAP) staining, revealing positive staining in HMFs and CAFs co-cultured with MDA-MB-231s, CAFs monoculture but not HMFs monoculture. Therefore, these results indicate that MDA-MB-231s are activating the HMFs. Interestingly, these results are in agreement with a recently published paper in which is demonstrated that MDA-MB-231 conditioned media activates normal dermal fibroblasts, resulting in the increased expression FAP among other fibroblast activation markers [46]. Finally, similar results have been achieved in a 3D environment in other types of cancers [39,57].

After the characterization, cancer cells were seeded in the different matrix compositions (i.e., fibronectin-rich collagen or collagen alone) with embedded fibroblasts (i.e., CAFs or HMFs) for 48 h. We observed that the number of MDA-MB-231 cells migrated into the matrix was significantly higher for the fibronectin-rich matrix, both with CAF and HMF, as compared to the collagen-only control. When we evaluated the average migration distance for the collagen-only matrices, cancer cell migration increased in the cocultures with CAFs, as compared to the co-cultures with HMFs. Interestingly, in the presence of HMFs, a normal component of the TME, fibronectin induces changes in the fibroblasts that, in turn, enhance the migration of the cancer cells. These results suggest that within a normal microenvironment (HMFs), a fibronectin-rich matrix is acting as a tumor-promoting factor, “educating” the normal environment to support cancer progression. On the other hand, we did not observe this effect in the presence of CAFs. However, it is known that CAFs secrete more fibronectin than HMFs, as demonstrated in Figure 2f,g and Figure S1e. Therefore, it is possible that the addition of fibronectin to promote tumor progression in the presence of CAFs does not make a difference. This increased fibronectin secretion in CAFs as compared to HMFs was corroborated in our system via Western Blot.

In breast cancer, tumor progression and metastasis have been found to be driven by matrix metalloproteinases (MMPs) shaping of the TME [58,59]. In this regard, in vitro studies have revealed that cancer cells induce stromal fibroblasts to express MMPs (i.e., MMP-9) [60], demonstrating the complex tumor-stromal crosstalk that occurs in breast cancer. Additional studies have found that a subset of the MMPs (e.g., MMP-2 and MMP-9) are upregulated in breast cancers and are associated with poor outcomes [61]. In addition, other MMPs (e.g., MMP-3), have been found to not only promote matrix degradation but to act as a signaling molecule that regulates mammary stem cell formation [62]. As expected, MMP secretion increased in the co-cultures as compared with the monocultures. Further, our results show that the presence of a fibronectin-rich matrix drives MMP secretion from HMFs but did not affect the secretion of MMPs from CAFs. These results support our hypothesis that the fibronectin-rich matrix-induced changes in the normal component (HMFs), such as an increase in MMP secretion, that allows cancer cells to migrate further. Overall, these results suggest that the matrix composition stimulates the normal component of the tumor microenvironment to secrete more MMPs, promoting cancer cell migration in normal microenvironments. However, another possible mechanism by which we observe an increase in cancer cell migration in the fibronectin-rich matrix could be due to remodeling of the ECM by the contractility of the HMFs. As has been demonstrated, in the presence of fibronectin, fibroblasts remodel the matrix, contributing to matrix alignment and in turn, promoting invasion in cancer cells [55].

It is well recognized that fibroblasts can interact and communicate with the surrounding ECM, resulting in ECM structure remodeling. The remodeling of the ECM is regulated, in part, through the secretion of matrix-degrading enzymes (i.e., MMPs), which are known to facilitate cancer invasion through degradation of the ECM. Therefore, we next studied the presence of collagen disruptions in the collagen using second harmonic generation imaging. This study revealed an increase in the distribution of matrix gap areas in fibronectin-rich matrices, where HMFs were co-cultured with cancer cells. This effect is consistent with the increase in MMP-2, -3, and -9 secretion in previous figures. However, the gap area in the co-culture with CAFs did not change in the fibronectin-rich matrix as compared with the collagen matrix. Interestingly, these MMPs can degrade fibronectin and not collagen I, suggesting that the fibronectin matrix is signaling the HMFs to produce MMPs that can degrade the surrounding matrix. As for the degradation of collagen I, these MMPs have the potential to activate other MMPs to degrade collagen I. 

To investigate if the degradation of the matrix was responsible for changes in MDA-MB-231 migration, we used a broad-spectrum MMP inhibitor: Marimastat. Marimastat inhibits MMP-2, MMP-9, MMP-1, and other MMPs and, it has been reported to inhibit cancer cell migration 3D in vitro at a 30 µM concentration [41] and to inhibit fibroblast mediated collagen hydrogel contraction at a 10 µM concentration [42]. In our model, marimastat treatment was not effective at reducing cancer cell migration or reducing the matrix gap area in the co-cultures with HMFs. Our results demonstrating that marimastat was not effective in the presence of normal fibroblasts, could provide an explanation of differences between the successful MMP inhibitors in vitro results, and in vivo the unsuccessful results in clinical trials. However, we cannot rule out the possibility that fibroblast tumor cell crosstalk or cues from ECM composition affect the secretion of additional factors from fibroblasts that drive cancer cell migration even in the presence of marimastat. Therefore, this hypothesis led us to investigate the influence of MMPs inhibitors using our model but in MDA-MB-231 monocultures.

Interestingly, MDA-MB-231 monoculture and in co-culture with CAFs showed the largest migration distance. Therefore, we hypothesized that migration must occur in a mechanism independent of MMP-2, -3, and -9 in these conditions. These results do not match the previously described results, which demonstrated the efficacy of the inhibitor in vitro [41,63]. However, previous studies were performed in cancer cells monocultures and did not consider the contribution of fibroblasts, the major MMP secreting cell. Previous in vitro results have led to intensive efforts to develop and translate broad-spectrum MMP inhibitors, such as marimastat for cancer treatment, which concluded due to disappointing results in multiple clinical trials [64]. Of relevance in breast cancer, a phase III trial of the MMP inhibitor marimastat in metastatic breast cancer found no therapeutic benefit [65]. MMP inhibitor treatments provided no benefit in early stages either, as demonstrated by a phase II trial of marimastat and rebimastat. This study found a high incidence of musculoskeletal toxicity and failure of chronic dose levels to maintain plasma levels within the target range for these drugs [66,67]. Our results indicate that in monocultures, the MMP inhibitors are highly effective, which are in agreement with the literature [41,68]. However, in co-culture, these doses may fall short in inhibiting fibroblast-secreted MMPs. Our results demonstrate the importance of in vitro models that incorporate TME components that are essential in the microenvironment. The best examples are previous in vitro studies demonstrating the efficacy of MMP inhibitors without incorporating fibroblasts, a highly MMP-secreting cell type [41,69]. The lack of incorporation of this component could explain the limited successes of MMP inhibitors in clinical trials. Due to the poor performance in clinical trials, investment toward MMP inhibitors has fallen short. However, basic researchers support the idea of using more selective inhibitors. For this reason, as future directions, the use of patient-specific cells to build relevant in vitro models can be useful to elucidate the specific MMP to be targeted.

Altogether, our microfluidic 3D in vitro model allowed us to mimic important features of the breast tumor microenvironment by including only a few key components of the tumor microenvironment. Using this model, our findings demonstrate the utility of our model to study the contribution of different microenvironment components (e.g., cellular or matrix components) on breast cancer cell migration. The usefulness of this model could be further improved by incorporating different cell types representing distinctive types of cancers, therefore providing researchers with a powerful tool to advance cancer research. As an example, triple-negative breast cancer (TNBC), a highly aggressive and migratory cancer type was represented in this study by using the cell line MDA-MB-231. Therefore, the incorporation of cell lines that represent the different molecular subtypes of breast cancer using this model could provide further insights into cancer progression. Further, we could test the relevance of this model by including other cancer types in the model and by using patient-derived cancer cells. In the future, we envision the current platform as a screening tool to test the influence of other matrix proteins that are found in vivo [19]. Overall, we presented a 3D in vitro model to recapitulate aspects of the breast tumor microenvironment. Using this model, we investigated the migration of cancer cells in a 3D microenvironment that contains components found in vivo such as fibroblasts and relevant matrix composition.

## 4. Materials and Methods

### 4.1. Cell Culture

Human mammary adenocarcinoma cells MDA-MB-231 were selected for high invasiveness and their ability to metastasize in vivo [69]. For all experiments, MDA-MB-231s (ATCC) were stably transfected with turbo green fluorescent protein (GFP) to visualize and quantify cell migration. Immortalized human mammary fibroblasts, referred to as HMFs, were derived from the stromal vascular fraction of a reduction mammoplasty and were a kind gift from Dr. Lisa Arndt’s lab (University of Wisconsin, Madison, WI, USA). Carcinoma associated fibroblasts (CAFs) cells were initially obtained from the Kuperwasser lab (Tufts University, Medford, MA, USA) [70]. All cells were routinely cultured in high glucose DMEM (Gibco, 11965092, Grand Island, NY, USA) supplemented with 10% fetal bovine serum (FBS 97068-085, VWR, Radnor, PA, USA) and 1% penicillin/streptomycin (ThermoFisher, 15140-122, Grand Island, NY, USA) and were kept in a humidified incubator at 37 °C with 5% CO_2_. All cells were cultured to 90–95% confluency for all experiments, and the media was changed every 2–3 days.

### 4.2. Device Fabrication

LumeNEXT fabrication was performed as previously described [71]. Briefly, the microdevice consisted of 2 polydimethylsiloxane (PDMS) layers, which defined the microchamber; and a suspended PDMS rod, which was removed after polymerization of a hydrogel in the main chamber to create a tubular lumen structure. To fabricate both layers of the microdevice, a traditional soft lithography technique was used, in which the layers were spun using SU-8 (MicroChem, Y13273, Newton, MA, USA) to create the silicon master molds. Polydimethylsiloxane (PDMS, Dow Corning, Sylgard 184, Midlan, MI, USA) was mixed at a 10:1 base to curing agent ratio and poured over the SU-8 silicon master molds. Using the same PDMS mixture, PDMS rods were fabricated by filling up a 25 gauge (Fisher Scientific, 14-840-84, Pittsburg, PA, USA) hypodermic needle with PDMS. PDMS components were then baked at 80 °C for 4 h. After baking, the PDMS rods were extracted from the needles, yielding PDMS rods of 280 μm in diameter. The 2 layers were aligned, ethanol-bonded together, and the PDMS rods were placed into the microdevice chamber. Finally, the microdevice was oxygen plasma bonded (Diener Femto system) to a glass-bottom MatTek dish (MatTek Corporation, P50G-1.5-30-F, Ashland, MA, USA), following a general protocol. This process yielded arrays of 6 microdevices that were used as technical replicates in our study. The microdevices were sterilized using UV irradiation for 15–20 min for further use.

### 4.3. Organotypic Cell Culture

#### 4.3.1. LumeNEXT Device Preparation

To achieve maximum hydrogel adhesion to the device chamber, a 2-step coating of 2% poly(ethyleneimine) (PEI, Sigma-Aldrich, 03880, St Louis, MO, USA) diluted in deionized DI water for 10 min was loaded into the side ports. The PEI solution was aspirated, and 0.4% glutaraldehyde (GA, Sigma-Aldrich, G6257) diluted in deionized (DI) water was loaded into the side ports and incubated at room temperature for 30 min. During the GA incubation, the collagen solution was prepared on ice (refer to Section 4.3.2). After the 30-minute GA incubation, the microdevices were washed 3 times with sterile DI water to remove any GA excess. At this point, devices were ready to be loaded with the collagen solution. To minimize evaporation, sacrificial phosphate-buffered saline (PBS, Fisher scientific, BP3991) was added around the side of the MatTek dish.

#### 4.3.2. Extracellular Matrix Preparation and Loading into the Device

High-density rat-tail collagen type 1 (Corning, 354249, Oneonta, NY, USA; referred as collagen through the text) was diluted with 5X PBS (diluted with DI water from the 10× stock described before) and neutralized with 0.5 M NaOH (Fisher Scientific, S318) achieving a final concentration of PBS 1×, and a pH of 7.4. This mixture was diluted with fibrinogen (Sigma-Aldrich, F8630) and media to achieve a final concentration of 2.25 mg/mL. For the collagen solution containing fibronectin, fibronectin solution (Sigma-Aldrich, F1141) was added to a final concentration of 100 μg/μL in the collagen. For experiments with stromal cells in the matrix, HMFs or CAFs, a final concentration of 500 cells/μL was added to their respective collagen solution. Immediately after washing, 8 µL of collagen solution was loaded through the side ports and polymerized at room temperature for 10 min. Finally, a small droplet of media (5 μL) was placed on top of the side ports to prevent evaporation, and devices were transferred to 37 °C for 1 h to allow collagen to polymerize fully.

#### 4.3.3. Preparation of Collagen I Solution Containing MDA-MB-231 and Loading into the Device

After incubation, a droplet of media (5 μL) was added to the input port under sterile conditions. To remove the PDMS rod, the rod was pulled through the output port using isopropanol-sterilized tweezers. This procedure yielded a hollow lumen structure within the collagen matrix filled up with media and ready for cell loading. All fluid handling procedures were conducted with standard pipettes, uniquely enabled by passive pumping [72]. A 1.5mg/mL collagen solution containing MDA-MB-231s was prepared, as indicated in Section 4.3.2, with the addition of the MDA-MB-231s at a final concentration of 16,666 cells/μL. Then, the media from the lumen was aspirated, and 3 µL of collagen solution containing MDA-MB-231 was loaded through the input port. Collagen was polymerized as described in previous sections. After incubation, the collagen solution left in the output port was aspirated to remove the excess of cancer cells. Then, 20 μL media was added to the output port, and devices were transferred to the incubator for overnight incubation. The next morning, the media was replenished and refreshed every day.

### 4.4. Imaging of Cancer Cell Migration Distance

The GFP signal from the MDA-MB-231 was used to track cancer cell migration out of the lumen. For each device, Z-stack imaging was performed after 48 h of culture. Bright-field images were acquired at 4X using a Nikon TI^®^ Eclipse inverted microscope (Melville, New York, NY, USA) and processed using the National Institutes of Health ImageJ software. To analyze the migration distance, each Z-stack was Z-projected, and the MDA-MB-231 distance from the lumen was quantified using Image J [73]. 

### 4.5. Cytokine Secretion Assay

Multiplexed protein secretion analysis was performed on the cancer-fibroblasts co-cultures, cancer monocultures, and fibroblast monocultures in both types of matrix composition. The analysis was performed using the Magnetic Bead-Based Multiplex ELISA system MAGPIX (Luminex Corporation, Austin, TX, USA) using a Human MMP Magnetic Panel (3-Plex) (R&D Systems, FCSTM07-03, Minneapolis, MN, USA) as described elsewhere [74]. Collected media from 6 cultured vessels at 24 and 48 h was pooled to increase the sample volume in each cultured condition. Sample preparation and detection was performed following the manufacturer’s protocol. Data were collected with xPonent software (Luminex), and soluble factor concentrations in media were calculated using mean fluorescence intensities (MFI) by creating a standard curve for each analyte using a 5-parameter logistic (5-PL) curve fit in Graphpad Prism.

### 4.6. Matrix Visualization by SHG Imaging and Analysis

Collagen hydrogel structure was visualized by second harmonic (SHG) using a custom-built inverted multiphoton microscope (Bruker Fluorescence Microscopy, Middleton, WI, USA), as described previously [38]. The system consisted of a titanium:sapphire laser (Spectra-Physics, Insight DS-Dual), an inverted microscope (Nikon, Eclipse Ti, Melville, New York, NY, USA), and a Nikon Apo 40×/1.25 WI λS objective. Collagen fibers were excited using an 890 nm infrared laser and collected the emission at 450 nm. Three images were collected per device (from 4 different devices) at a 100 μm distance from the bottom of the gel, to avoid edge effects.

SHG images from the different collagen matrices were analyzed using the Diameter J plugin for ImageJ. Briefly, image segmentation was performed for each image following binarization. All gap areas in the image were automatically measured with this plugin.

### 4.7. Cell Tracker and Immunofluorescence Staining

For experiments in which cells were fluorescently labeled with red or blue cell tracker, stock solutions of cell tracker red CMTPX (Thermo Fisher, C34552) and blue (Thermo Fisher, C2110) were prepared following supplier instructions. Then, the stock solution was diluted 1:1000 in the growth medium. Cells were trypsinized and incubated in the cell-tracker diluted medium for 30 min. Finally, cells were washed twice with 1X PBS to remove the excess of the cell tracker.

For immunofluorescence staining, cells were washed with PBS for 30 min between each step. Unless specified otherwise, steps took place at room temperature. Washing buffer (0.1% PBS-Tween 80 (Sigma-Aldrich, P1754) and blocking buffer (3% Bovine Serum Albumin (BSA, Sigma-Aldrich, A9056) in 0.1% PBS-Tween 80) were made in advance and stored at 4 °C until use. Cells were fixed with 4% paraformaldehyde (PFA) (EMScience, 15700, Hatfield, PA, USA) for 15 min, then incubated with 0.2% Triton® X-100 (MP Biomedicals, 807426, Santa Ana, CA, USA) for 30 min for permeabilization. Finally, devices were incubated with 10 µL of blocking buffer at 4oC overnight. Texas Red-X Phalloidin (ThermoFisher Scientific, T7471, Waltham, MA, USA) and DAPI (ThermoFisher Scientific, D3571) were used to stain actin cytoskeleton and nuclei, respectively. An anti-fibronectin antibody was used to stain the presence of fibronectin in the collagen gel (Abcam, ab2413, Cambridge, UK). For activated fibroblasts characterization, an anti-fibroblast activation protein antibody (Abcam, ab53066) and an anti-podoplanin antibody (Abcam, ab11936). Fluorescent images were acquired at 10× using a Nikon TI Eclipse inverted microscope (Melville) and processed using the National Institutes of Health ImageJ software.

### 4.8. Immunoblots

Cell lysates from HMFs or CAFs that were cultured in a 3D collagen matrix were analyzed for the expression of fibronectin. To ensure adequate protein concentration for western blot analysis, 2 × 10^5^ cells were embedded in a final 1 mL volume of 3 mg/mL type-I collagen hydrogel. Cells were cultured for 72 h, gels were washed 3× with PBS prior to lysis in 2× RIPA [75] and 5× Laemmli sample buffer (62.5 mmol/L Tris pH 6.8, 20% glycerol, 2% SDS, 10% β-mercaptoethanol, 0.3125 mg/mL bromophenol blue) and boiled for 5 min. Western blot analysis was performed with Mini Gel Tank (Life Technologies, A25977, Carlsbad, CA, USA), and the bands were detected with Luminia Crescendo Western HRP Substrate (Millipore, WBLUR0100, Burlington, MA, USA). RUBY staining of the SDS-page gel was performed with SYPRO Ruby protein gel stain (Invitrogen, S12000, Carlsbad, CA, USA). Relative expression of fibronectin (anti-fibronectin (BD, BD Biologicals, 610077) was calculated by normalizing fibronectin intensity to the intensity of total protein stained by SYPRO Ruby.

### 4.9. MMP Inhibition

Marimastat (BB-2516, Selleckchem, S7156, Houston, TX, USA) was used to inhibit the activity of different MMPs, such as (MMP -1, 2, -7, -14). A stock solution was prepared at 10 mM concentration following supplier instructions. For the treatment with marimastat in the different microenvironments, the stock solution was diluted to a final concentration of 30 µM marimastat. Treatment with 30 µM Marimastat or vehicle control (DMSO) was performed for 48 h, following imaging acquisition. Image analysis was performed to determine changes in migration distance, as described in previous sections.

### 4.10. Statistical Analysis

All the experiments were repeated at least 3 times as independent biological replicates. All results were presented as the mean ± standard deviation of the mean. Data were analyzed using GraphPad Prism 8 (GraphPad Software, La Jolla, CA, USA), and statistical significance was set at *p* < 0.05. One-to-one comparisons were performed with an unpaired Student t-test with Welch’s correction (if SDs were inhomogeneous) after the normal distribution was proved via the Shapiro-Wilk test. If the normality test was not passed, a non-parametric test was performed (Mann-Whitney test).

## 5. Conclusions

The microfluidic model presented in this paper mimics the in vivo invasion of cancer cells within a 3D microenvironment that includes relevant fibroblasts and extracellular matrix composition. We found that a tumor-like matrix, such a fibronectin-rich matrix altered the normal mammary fibroblasts over the cancer-associated fibroblasts. Some of the changes observed were an increase in the number of migrating cells, longer migration distance, higher secretion of MMPs, as well as a resulting increase in matrix remodeling. In co-culture with HMFs, MMP inhibition was effective in inhibiting cancer cell migration in the collagen matrix and not in the fibronectin-rich matrix. In addition, MMP inhibition was performed in cancer cells monocultures and was found to be effective regardless of the matrix, which is consistent with the literature. In conclusion, without incorporating fibroblasts, a highly MMP-secreting cell type, MMPs inhibitors are highly effective. Therefore, the lack of incorporation of this component in in vitro studies could explain the limited successes of MMP inhibitors in clinical trials, which further suggests the need to develop alternate methods to specifically target fibroblast induced matrix remodeling. Examples of ongoing CAF anti-cancer therapy include CAF specific depletion with anti-FAP antibodies [76], maintaining CAFs in a quiescent state with Vitamin D or Retinoic Acid, [77] or targeting CAF signaling pathways involved in matrix remodeling [78]. Overall, this data demonstrates the capability of the model to pinpoint the contribution of different components of the TME. 

## Supplementary Materials

The following are available online at https://www.mdpi.com/2072-6694/12/5/1173/s1, Figure S1: Characterization of cancer cells and fibroblasts, Figure S2: Indirect co-culture using a modified 96-well plate (MicroDUO), Figure S3: MMP protein secretion in monocultures, Figure S4: Matrix visualization via Second Harmonic Generation (SHG) imaging in the MDA-MB-231 monoculture conditions, Figure S5: Effect of MMP inhibition on cancer cell migration in the MDA-MB-231 monoculture conditions, Figure S6: Matrix visualization via Second Harmonic Generation (SHG) imaging after MMP inhibition, Figure S7: Western blot of fibronectin in HMFs and CAFs cultured in HD gels used for Figure 2, Table S1: Densitometry raw data quantified from Western Blot shown in Figure 2, Methods for the assessment of deposited fibronectin by fibroblasts, Methods for indirect co-culture using a modified 96-well plate (MicroDUO).
