# Supplementary material for: Breast Fibroblasts and ECM Components Modulate Breast Cancer Cell Migration through the Secretion of MMPs in a 3D Microfluidic Co-Culture Model
Source: Cancers (Basel). 2020 May 6;12(5):1173. doi: 10.3390/cancers12051173 (PMC7281408; doi:10.3390/cancers12051173)
Supplement: Supplementary file 1 [file cancers-12-01173-s001.pdf]

cancers

MDPI

Article

Breast fibroblasts and ECM components modulate breast cancer cell migration through the secretion of MMPs in a 3D microfluidic co-culture model

Karina M. Lugo-Cintrón, Max M. Gong, José M. Ayuso, Lucas A.Tomko, David J. Beebe, María Virumbrales-Muñoz, and Suzanne M. Ponik

Supplementary Materials:

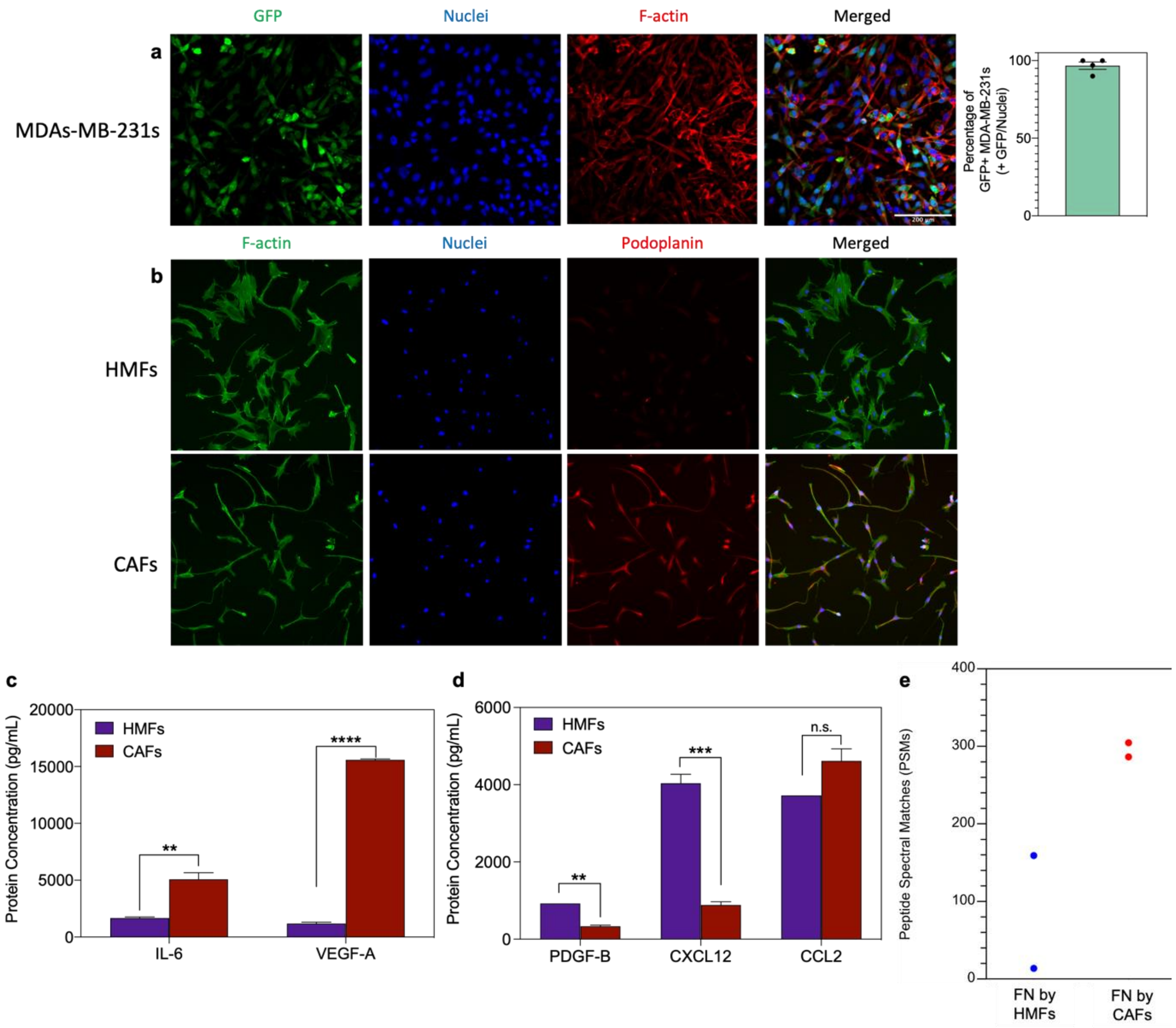

Figure S1. Characterization of cancer cells and fibroblasts. (**a**) MDA-MB-231 immunofluorescence. GFP (green), nuclei (blue) and F-actin (red). Scale bar = 200 µm. Percentage of GFP+ MDA-MB-231 graph. (**b**) HMFs and CAFs immunofluorescence. F-actin (green), nuclei (blue) and podoplanin (red). (**c**,**d**) Fibroblasts protein secretions. (**e**) Fibronectin deposition by fibroblasts.

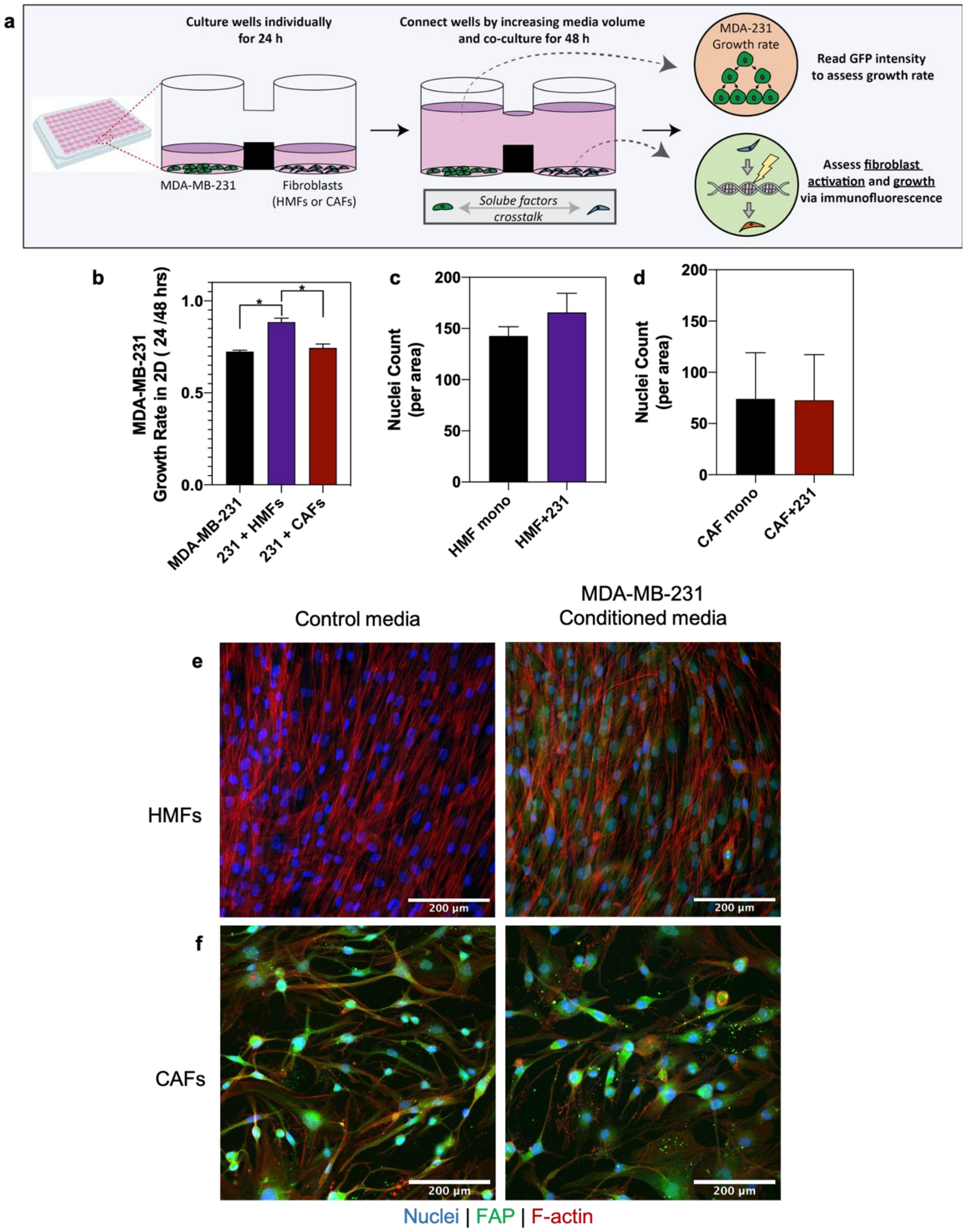

Figure S2. Indirect co-culture using a modified 96-well plate (MicroDUO). (**a**) Schematic of the process in 2D. (**b**) MDA-MB-231 growth rate in 2D (**c**,**d**) Fibroblasts nuclei count (**e**,**f**) HMFs and CAFs immunofluorescence in monoculture and co-culture with MDA-MB-231. F-actin (red), nuclei (blue) and FAP (green). Scale bar = 200 μm. * $p < 0.05$.

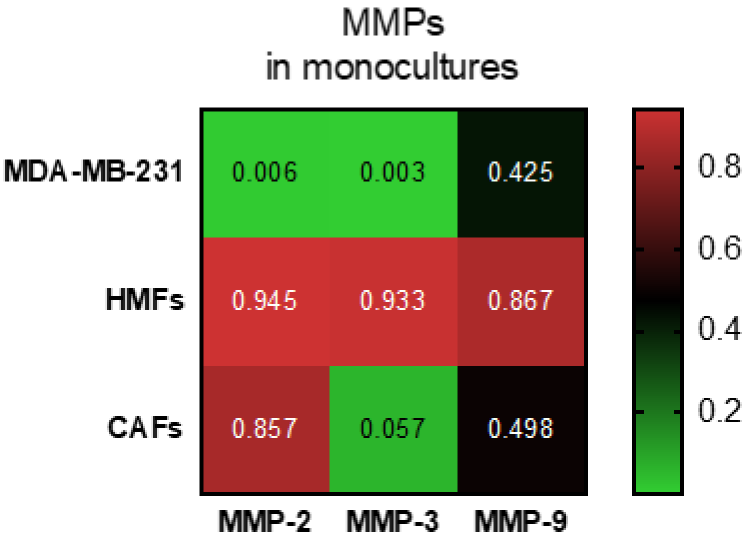

Figure S3. MMP protein secretion in monocultures**.** Heat-map showing MMP-2, -3 and -9 secretion in the HMFs and CAFs monocultures compared to the MDA-MB-231 monoculture. Heat-map represents the average of at least 4 individual devices.

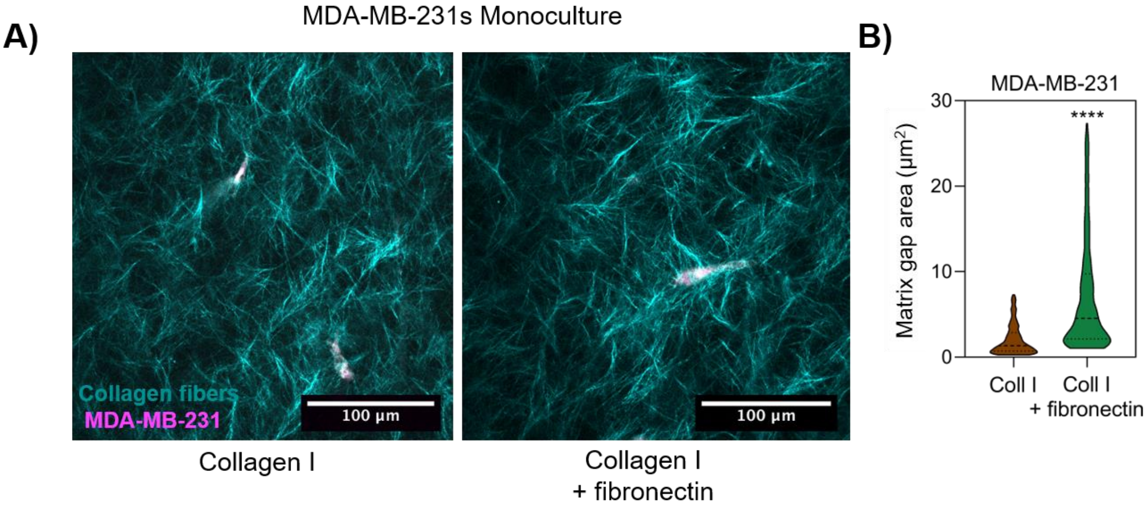

Figure S4. Matrix visualization via Second Harmonic Generation (SHG) imaging in the MDA-MB-231 monoculture conditions**.** (**A**) Collagen fibers are depicted in cyan, whereas MDA-MB-231cells appear in magenta. Left: Collagen matrix and Right: fibronectin-rich matrix. (**B**) Quantification of matrix gap area. Violin plot represents the distribution of the data with the average and SD of at least 4 individual devices. ****$p \leq 0.05$.

.

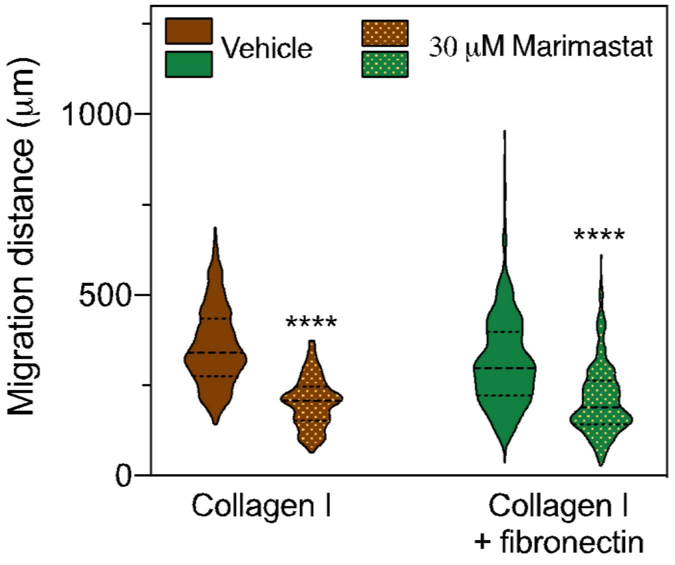

Figure S5. Effect of MMP inhibition on cancer cell migration in the MDA-MB-231 monoculture conditions. Monocultures within the different matrices were treated for 48 hours with 30 μM marimastat (MMP inhibitor) and a DMSO vehicle (control). Migration distance of MDA-MB-231 in the collagen matrix (left) and the fibronectin-rich matrix (right) for the vehicle control and treatment. Violin plot represents the distribution of the data with the average and SD, n at least 4 individual devices. **** $p < 0.0001$.

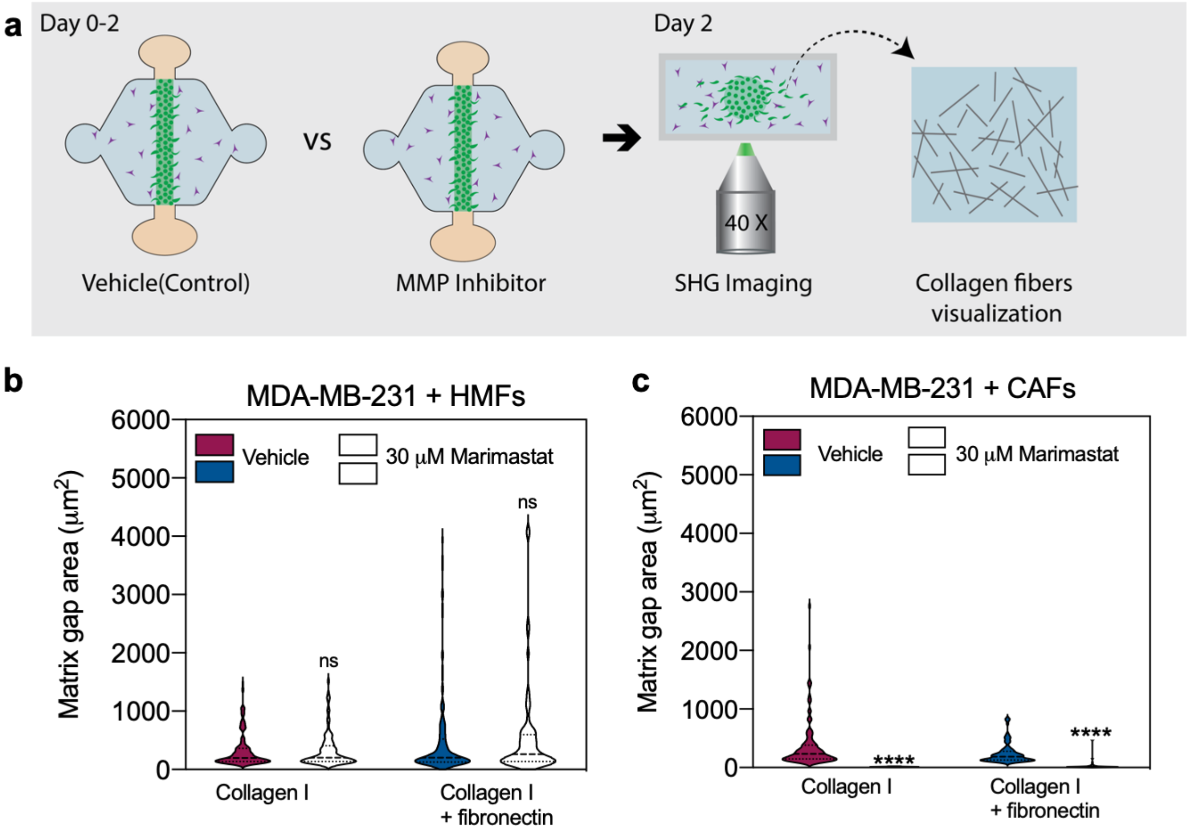

Figure S6. Matrix visualization via Second Harmonic Generation (SHG) imaging after MMP inhibition. (**a**) Schematic of the process. (**b**,**c**) Quantification of matrix gap area. The Violin plots

represent the distribution of the data with the average and SD, n = at least 4 individual devices. **** $p < 0.0001$.

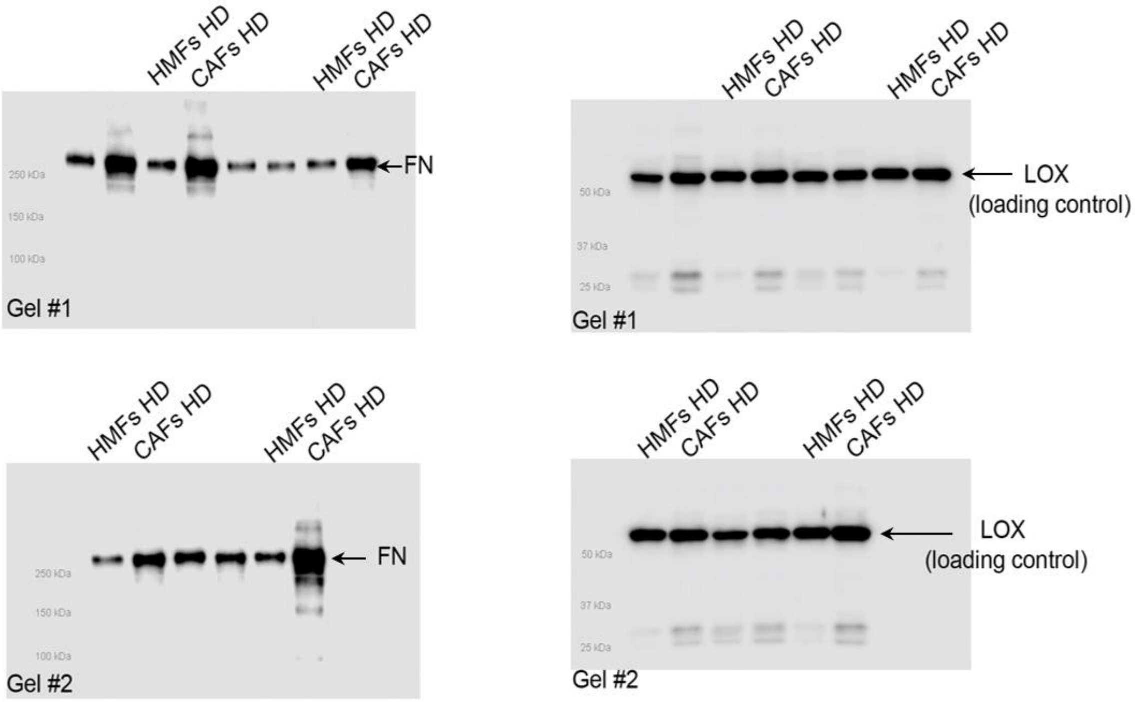

Figure S7. Western blot of fibronectin in HMFs and CAFs cultured in HD gels used for Figure 2. Gels used for fibronectin (FN) densitometry quantification (Left) and Lysyl Oxidase (LOX; used as loading control to demonstrate equal protein loading). Total protein was quantified by BCA assay and 30ug of protein loaded per well. Relevant lanes have been annotated as HMFs and CAFs in High density gels (HD).

Table S1. Densitometry raw data quantified from Western Blot shown in Figure 2.

**Signal from Fn**		**Protein Loading Control**		**Fn Signal/ Loading Control Signal**	
HMF	*CAF*	*HMF*	*CAF*	*HMF*	*CAF*
0.402	1.71	40.1	35.6	1.00	4.80
0.308	0.888	46.0	49.9	0.667	1.78
0.304	0.957	52.2	41.2	0.582	2.32
0.462	2.12	53.2	70.3	0.868	3.01

Supplementary Methods:

1. *Assessment of deposited fibronectin by fibroblasts*

To generate matrix for study, fibroblasts were grown to confluency in 6-well cluster plates (Corning Costar, #3506) and the media was supplemented with 50mg/mL ascorbic acid (Sigma, cat. no. A544-25G) to a final concentration of 50μg/mL. Fibroblasts were maintained at confluency for 10–14 days, with 50% media changes every 2–3 days. After the culturing period, fibroblasts' media was removed and the cells were rinsed in warmed PBS. Then, approx. 500μL of alkaline fibroblast lysis buffer (previously described[1]), supplemented with proteinase and phosphatase inhibitors (NaF, ortho-vanadate, and proteinase inhibitor cocktail ref), was then added to each well and allowed to incubate, while rocking, at room temperature for 10 min. After removing the supernatant, the remaining ECM in each well was incubated O/N at RT in 8M urea to solubilize the ECM prior to mass spec analysis (completed in collaboration with Dr. Kirk Hansen).

2. Indirect co-culture using a modified 96-well plate (MicroDUO)

MDA-MB-231s and fibroblasts (HMFs or CAFs) were seeded in the co-culture well-plate (MicroDUO, Onexio Biosystems, LLC, Madison, WI,USA). For co-culture experiments, cells were seeded in adjacent wells at 5000 cells/well for the fibroblasts and 30,000 cells/well for the cancer cells. After 24 h of culture, culture media was added, connecting both wells and allowing paracrine signaling for 48 h. Then, GFP signal from the MDA-MB-231s was quantified with a ClarioStar machine. GFP intensity signal can be coorelated with MDA-MB-231 growth rate, as characterized by us (data not shown). Finally, cells were fixed and stained.

Supplementary References

1. Yang, N.; Mosher, R.; Seo, S.; Beebe, D.; Friedl, A. Syndecan-1 in breast cancer stroma fibroblasts regulates extracellular matrix fiber organization and carcinoma cell motility. *The American journal of pathology* **2011**, *178*, 325–335.
